# Time-course variation of statistics embedded in music: Corpus study on implicit learning and knowledge

**DOI:** 10.1371/journal.pone.0196493

**Published:** 2018-05-09

**Authors:** Tatsuya Daikoku

**Affiliations:** Department of Neuropsychology, Max Planck Institute for Human Cognitive and Brain Sciences, Leipzig, Germany; University of Manchester, UNITED KINGDOM

## Abstract

Learning and knowledge of transitional probability in sequences like music, called statistical learning and knowledge, are considered implicit processes that occur without intention to learn and awareness of what one knows. This implicit statistical knowledge can be alternatively expressed via abstract medium such as musical melody, which suggests this knowledge is reflected in melodies written by a composer. This study investigates how statistics in music vary over a composer’s lifetime. Transitional probabilities of highest-pitch sequences in Ludwig van Beethoven’s Piano Sonata were calculated based on different hierarchical Markov models. Each interval pattern was ordered based on the sonata opus number. The transitional probabilities of sequential patterns that are musical universal in music gradually decreased, suggesting that time-course variations of statistics in music reflect time-course variations of a composer’s statistical knowledge. This study sheds new light on novel methodologies that may be able to evaluate the time-course variation of composer’s implicit knowledge using musical scores.

## 1. Introduction

### 1.1. Implicit learning and statistical learning

Implicit learning and knowledge is a ubiquitous phenomenon that occurs without the intention to learn and awareness of what we know [1,2]. A number of studies have investigated this form of learning using several paradigms [3,4, 5,6]. In a series of psychological and neurological studies, the learning and knowledge of transitional probability in sequential information such as music and language—called statistical learning and statistical knowledge—have been considered implicit processes that could be performed domain-generally in both music and language regardless of sensory modalities. Thus, the terms implicit learning and statistical learning have been used interchangeably and are regarded as the same phenomenon [6], although some researchers stated statistical knowledge and implicit knowledge may exactly not be same thing. In daily life, using the framework of statistical learning, we benefit immensely from artificial intelligence such as machine learning that gives computers learning ability similar to humans [7,8]. By calculating statistics on what we did in the past, computers can predict what we will do next, and they also give the best information, even when we are unaware of what we statistically want. In several fields of study on machine learning and natural language and music processing [9–18], the Markov chain has often been used as a model of the artificial grammar of language and music. The Markov chain, which was first reported by Markov [19], is a mathematical system in which the probability of the forthcoming state is statistically defined only by the latest state. The use of the Markov chains embedded in tone sequences allows us to verify the statistical structure of music [12–18] and statistical learning and knowledge in humans [20–23].

There are a number of evidences suggesting that statistical knowledge is implicit, although some researchers report that knowledge gained from statistical learning may result in both implicit and explicit knowledge [24]. Due to the implicitness of statistical learning in humans, however, we cannot specify the learned statistical knowledge, even when the learning effects are neurophysiologically unveiled [21,25,26] based on the framework of predictive coding [27]. In other words, implicit statistical knowledge can be acquired in our brains even though it does not reach the level of explicit awareness of what we learned. Despite the fact that humans are unaware of the learned statistical knowledge of auditory sequences, they can, however, identify that sequences containing high transitional probabilities sound familiar [20,21]. That is to say, although statistical knowledge itself may be implicit to a certain degree and does not reach the level of explicit awareness, it can be alternatively expressed via an abstract medium such as a musical melody [12–18].

### 1.2. Characteristics of music: Implicit and explicit aspects

Music has numerous domain-specific structures (e.g., isochronic metrical grids, tonal pitch spaces, hierarchical tension, and attraction contours based on the structure of a melody and chord progression), and cannot be fully explained solely by the domain-general statistics of transitional probabilities [28,29]. Thus, the general hypothesis is that music is learned by both statistical learning and music-specific learning [30,31]. In general, musical composers explicitly learn music-specific knowledge such as musical grammar, and intentionally follow these frameworks when composing music. Musical rules and how they should be followed vary over time and among traditions, genres, and composers themselves. Therefore, the characteristics of music can be extracted based on music-specific structures, such as the harmony, tonalities, relative pitches, and musical intervals in the musical scores [32–42]. To the best of our knowledge, however, few studies have verified whether the characteristics of music can be extracted based on the statistical structure in the musical scores. Furthermore, no study has investigated how the characteristics of the statistical structure in music varies over a composer’s lifetime.

### 1.3. Informatics approach of musical implicit learning

According to the previous studies, musical creativities in part depend on statistical learning [43], and the statistical distribution of music could partly represent composer’s statistical knowledge [12–18]. That is, in the framework of human statistical learning theory and information theory, a higher-probability sequence may represent one that a composer is more likely to choose compared to lower-probability sequence. Indeed, informatics approach is often used to understand general music acquisition [33–42] and the mental representation of implicit knowledge [44–46]. Particularly, PARSER [47], Competitive Chunker [48], Information Dynamics of Music (IDyOM) [12,15], and n-gram models [49] underpin the hypothesis that music is acquired based on statistical distribution of music. Rohrmeier and colleague also applied neural network such as simple recurrent network (SRN) to human’s implicit learning model [50]. They showed that strong learning effect for n-gram models and a weaker effect for SRN, partly suggesting that SRN and nGram (i.e., transitional probability based on Markov chain) models may represent the ability of general humans and musical experts, respectively. Thus, the time-course variations of statistical structures in music may represent the time-course variations of a professional composer’s statistical knowledge. Nevertheless, there are few studies that investigate time-course variations of a composer’s statistical knowledge by analyzing musical score. To understand mental representation of statistical knowledge in music, it is important to examine the relationships among statistical structures in music, music composition, and implicit knowledge.

### 1.4. Musicological aspects on the statistics of Ludwig van Beethoven’s Piano Sonata

In the transition from eighteenth-century musical classicism to nineteenth-century romanticism in Western music, Ludwig van Beethoven was a German composer and pianist (1770–1882) who was highly influential to other composers. In musicological study, Beethoven’s compositional career is divided into early (around 1802), middle (around 1802 to 1814), and late periods (from about 1814) [51,52]. Compared to his works in the early period, which were strongly influenced by his predecessors in classicism, such as Wolfgang Amadeus Mozart (1756–1792) and Franz Joseph Haydn (1732–1809), his works in the late period, when he was already troubled by deafness and irritability brought on by chronic abdominal pain, were considered to show his personal expression and intellectual depth [51,52]. It is also believed that Beethoven always explored new directions and gradually expanded his scope of music over his lifetime [51,52]. Musicological researchers suggest that Beethoven’s music expressed his vision of life [53,54]. Sullivan [53] claimed that Beethoven’s vision was the product of his character and experience. Thus, his psychological variations on thinking and experience may form the character of his music [55]. The present study hypothesized that the time-course variations of statistical structures in Beethoven’s music over his lifetime might reflect the time-course variations of his statistical knowledge. As such, it would be very interesting to examine if the psychological variations in which Beethoven explored new directions and gradually expanded his scope of music over his lifetime [51,52] were reflected in the statistics of his music. Based on the hypothesis, the present study investigated how statistical structures in music vary over a composer’s lifetime.

### 1.5. Study purpose

The present study aimed to investigate how statistical structures in music vary over a composer’s lifetime. The transitional probabilities of the sequences containing the highest pitches in Ludwig van Beethoven’s Piano Sonata with all of the movements (Piano Sonata No.1 in F minor, Op.2-1 to No.32 in C minor, Op.111) were calculated based on seven different hierarchical Markov stochastic models (i.e., first- to seven-order Markov chains). Although melody is sometimes not highest pitches e.g. bass melodies), the present study only analyzed the highest pitch because the definition of melody in each title of music is still controversial in musicological study, different melodies could concurrently appear in some titles of music, and melody is often played in highest pitches. To understand how the transitional probability of more general sequences that is consistently used by one composer, the present study just targeted the interval pattern that appear in all 32 sonatas. The time-course variations of the transitional probabilities in each interval pattern that appear in all 32 sonatas were examined. It was hypothesized that there may be two types of time-course variations: transitional probabilities that gradually decrease and those that gradually increase, consistent with the sonata opus number. If so, these findings suggest that the statistical knowledge of music gradually shift over a composer’s lifetime.

## 2. Methods

The Ludwig van Beethoven’s Piano Sonata and all of its movements (No.1 in F minor, Op.2-1 to No.32 in C minor, Op.111, composed 1795–1822) was used in the present study (Beethoven, Piano Sonata No.1 to No.32, Breitkopf & Härtel, Leiptig). Using a scorewriter (Finale version 25, MI Seven Japan, Inc.), electronic scoring data of the sequences of highest pitch were extracted from the XML files. The highest pitches were chosen based on the following definitions: the highest pitches that can be played at a given point in time, the pitches with slurs can be counted as one, and the grace notes were excluded (S1 Table). Using all the sequences of highest pitches in a movement of a Sonata, the transitional probabilities of the sequences of highest pitches were calculated as a statistic based on Markov chains. The weighted averages of transitional probabilities of all the movements in a Piano Sonata were calculated. The probability of a forthcoming tone was statistically defined by the last tone to seven successive tones, respectively (i.e., first- to seven-order Markov chains).

*n*^-^order Markov model is based on the conditional probability of an element e_i+1_, given the preceding *n* elements:

$$P(e_{n+1}|e_{n})=P\left(e_{n+1}\cap e_{n}\right)/P\left(e_{n}\right)$$ (1)

**Fig 1:**
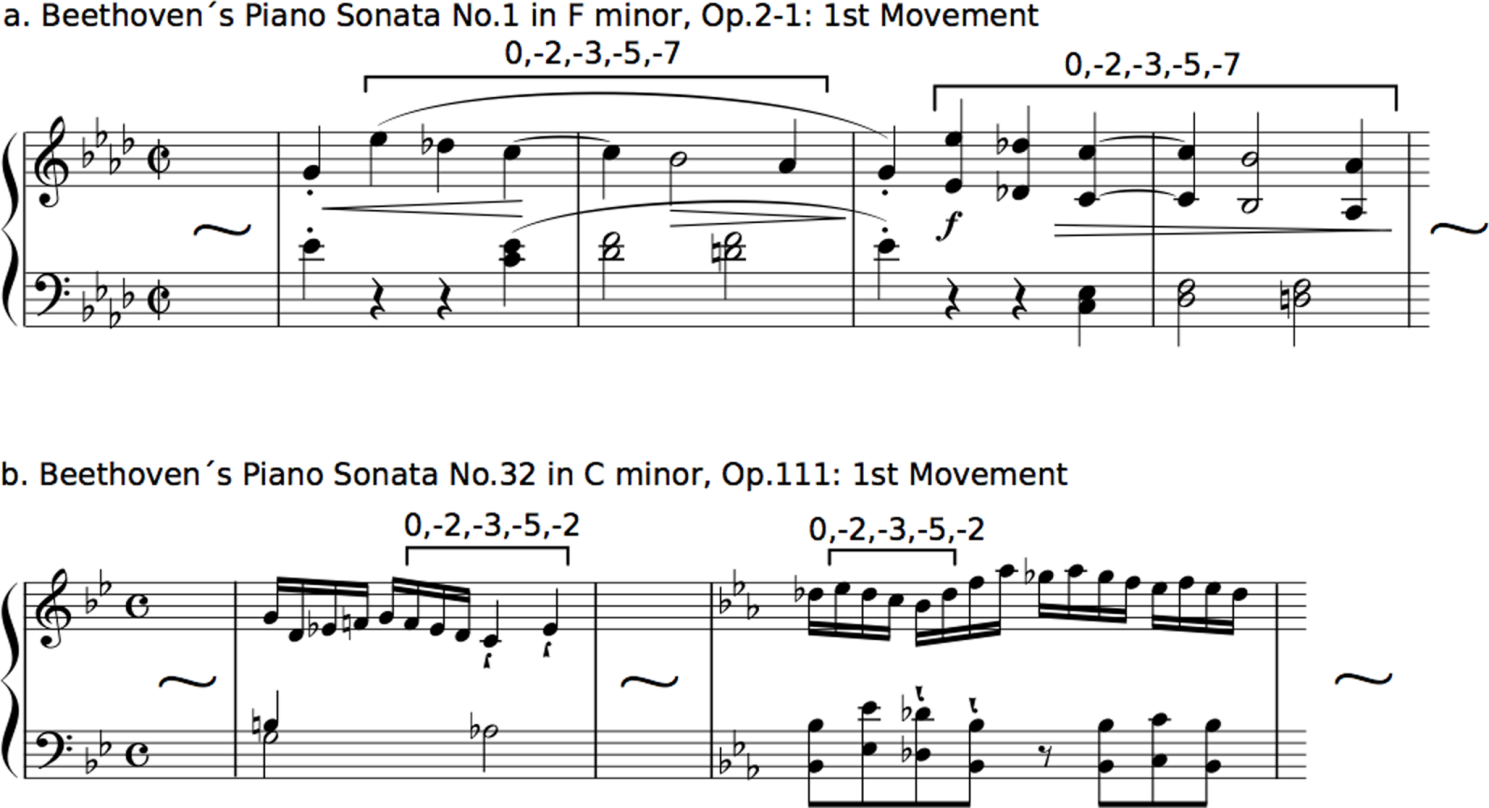
**Representative transitions of [0,–2,–3,–5,–7] in Beethoven’s Piano Sonata No.1 in F minor, Op.2-1 (a), and those of [0,–2,–3,–5,–2] in Beethoven’s Piano Sonata No.32 in C minor, Op.111 (b).** Based on the fourth-order Markov chain, the forthcoming states with the highest transitional probability defined by the last four states of [0, –2, –3, –5] are -7 in No.1 and -2 in No.32, respectively (see Table 10).

Then, for each type of pitch transition, all pitches were numbered so that the first pitch was 0 in each transition, and an increase or decrease in a semitone was 1 and -1 based on the first pitch, respectively. The representative examples were shown in Fig 1. This revealed interval patterns but not pitch pattern [43,56]. This procedure was employed to eliminate the effects of the change of key on interval patterns. The interpretation of the change of key depends on musicians, and it is difficult to define in an objective manner. Thus, the results in the present study may represent a variation of statistics associated with relative pitch rather than absolute pitch.

Each interval pattern was ordered based on the Sonata opus numbers (i.e., Piano Sonata No.1 in F minor, Op.2-1 to No.32 in C minor, Op.111). Using the interval patterns that appear in all 32 sonatas, the time-course variations of the transitional probabilities were analyzed by multiple regression analyses using the stepwise method. Statistical significance levels were set at *p* = 0.05 for all analyses. The criteria of the variance inflation factor (VIF) and condition index (CI) were set at VIF < 2 and CI < 20 to confirm that there was no multi collinearity [57]. Based on the stepwise method, the regression model that showed greatest numbers of interval patterns were adopted. Next, all of the highest-order transitional probabilities that showed statistical significance were calculated in the first (No.1) and last (No.32) sonatas. Furthermore, the representative phrases that transitioned with the highest probability in the first and last sonatas were decoded as music scores.

## 3. Results

Twenty-two interval patterns with two tones that appear in all 32 sonatas were detected ([0,–1], [0,–12], [0,–2], [0,–3], [0,–4], [0,–5], [0,–6], [0,–7], [0,–9], [0,0], [0,1], [0,12], [0,2], [0,3], [0,4], [0,5], [0,6], [0,7], [0,8], and [0,9]) (Table 1). Using these interval patterns, a multiple linear regression based on the stepwise method was carried out to predict the sonata opus numbers (i.e., Piano Sonata No.1 in F minor, Op.2-1 to No.32 in C minor, Op.111), based on the transitional probabilities of the 22 interval patterns in the first-order Markov chain. A significant regression equation was found (F(2,29) = 5.96, *p* = 0.007), with an adjusted R^2^ of 0.24 (Table 2). The predicted sonata opus number is equal to 18.56–137.64 (transition of [0,1]) + 147.308 (transition of [0,2]). The transitional probabilities of [0,1] and [0,2] gradually decreased and increased consistently with the ascending order of the sonata opus numbers, respectively ([0,1] *p* = 0.007, [0,2] *p* = 0.021). These transitional probabilities were significant predictors of the sonata opus numbers.

**Table 1:** Transitional probabilities calculated using first-order Markov chains for each of the interval patterns.

Op.	Interval pattern																			
	0,-12	0,-9	0,-7	0,-6	0,-5	0,-4	0,-3	0,-2	0,-1	0,0	0,1	0,2	0,3	0,4	0,5	0,6	0,7	0,8	0,9	0,12
**1**	0.004	0.008	0.017	0.011	0.032	0.032	0.078	0.180	0.147	0.096	0.104	0.092	0.051	0.022	0.034	0.006	0.021	0.023	0.015	0.004
**2**	0.016	0.006	0.007	0.006	0.019	0.029	0.050	0.175	0.120	0.090	0.164	0.101	0.045	0.031	0.033	0.007	0.019	0.013	0.008	0.019
**3**	0.029	0.013	0.015	0.009	0.025	0.034	0.064	0.125	0.116	0.042	0.122	0.082	0.044	0.037	0.037	0.010	0.015	0.009	0.021	0.067
**4**	0.032	0.014	0.014	0.017	0.022	0.030	0.056	0.120	0.100	0.071	0.119	0.072	0.084	0.048	0.040	0.022	0.019	0.020	0.015	0.018
**5**	0.026	0.004	0.006	0.008	0.026	0.028	0.050	0.163	0.131	0.118	0.100	0.074	0.056	0.019	0.033	0.018	0.027	0.019	0.015	0.033
**6**	0.027	0.022	0.019	0.005	0.030	0.025	0.039	0.123	0.096	0.160	0.095	0.078	0.047	0.018	0.027	0.007	0.018	0.006	0.013	0.080
**7**	0.013	0.007	0.016	0.008	0.031	0.042	0.040	0.151	0.138	0.074	0.144	0.067	0.040	0.046	0.032	0.009	0.013	0.016	0.022	0.022
**8**	0.050	0.006	0.016	0.006	0.021	0.025	0.053	0.150	0.134	0.081	0.111	0.090	0.047	0.027	0.042	0.009	0.020	0.012	0.009	0.029
**9**	0.012	0.006	0.011	0.017	0.034	0.046	0.049	0.143	0.132	0.100	0.148	0.076	0.052	0.032	0.043	0.010	0.010	0.018	0.014	0.014
**10**	0.003	0.009	0.015	0.013	0.027	0.043	0.055	0.154	0.099	0.100	0.179	0.129	0.020	0.009	0.017	0.006	0.015	0.010	0.016	0.031
**11**	0.010	0.007	0.013	0.017	0.029	0.030	0.092	0.140	0.108	0.036	0.153	0.087	0.046	0.029	0.031	0.013	0.012	0.016	0.019	0.047
**12**	0.016	0.021	0.020	0.014	0.030	0.024	0.077	0.100	0.075	0.160	0.083	0.073	0.060	0.037	0.060	0.012	0.015	0.022	0.024	0.015
**13**	0.005	0.013	0.018	0.019	0.054	0.051	0.098	0.109	0.084	0.093	0.080	0.067	0.059	0.026	0.033	0.014	0.019	0.021	0.049	0.006
**14**	0.010	0.023	0.035	0.012	0.048	0.029	0.043	0.074	0.052	0.118	0.052	0.069	0.111	0.061	0.082	0.020	0.027	0.022	0.014	0.018
**15**	0.020	0.002	0.007	0.002	0.019	0.033	0.061	0.182	0.156	0.075	0.112	0.059	0.065	0.025	0.029	0.007	0.014	0.010	0.017	0.038
**16**	0.034	0.003	0.013	0.013	0.034	0.046	0.057	0.142	0.115	0.091	0.131	0.080	0.059	0.049	0.029	0.008	0.008	0.016	0.007	0.037
**17**	0.039	0.017	0.017	0.015	0.028	0.028	0.060	0.064	0.113	0.098	0.116	0.041	0.070	0.041	0.038	0.018	0.017	0.023	0.014	0.063
**18**	0.013	0.011	0.024	0.009	0.046	0.042	0.079	0.088	0.061	0.124	0.119	0.082	0.093	0.054	0.042	0.004	0.020	0.012	0.011	0.007
**19**	0.002	0.010	0.012	0.016	0.018	0.025	0.041	0.161	0.139	0.126	0.147	0.108	0.038	0.027	0.040	0.004	0.018	0.012	0.007	0.014
**20**	0.001	0.015	0.007	0.004	0.024	0.024	0.046	0.193	0.081	0.169	0.049	0.096	0.036	0.019	0.018	0.007	0.018	0.010	0.017	0.005
**21**	0.017	0.005	0.014	0.012	0.045	0.062	0.110	0.121	0.095	0.060	0.102	0.071	0.082	0.050	0.045	0.008	0.012	0.010	0.011	0.026
**22**	0.019	0.022	0.072	0.026	0.017	0.030	0.057	0.058	0.053	0.071	0.087	0.067	0.053	0.045	0.049	0.048	0.034	0.036	0.042	0.027
**23**	0.025	0.012	0.012	0.012	0.042	0.048	0.086	0.122	0.094	0.084	0.066	0.066	0.107	0.062	0.054	0.013	0.016	0.015	0.014	0.022
**24**	0.005	0.015	0.025	0.044	0.036	0.041	0.084	0.108	0.096	0.064	0.097	0.078	0.076	0.030	0.043	0.008	0.018	0.019	0.037	0.025
**25**	0.015	0.022	0.034	0.010	0.052	0.066	0.070	0.124	0.069	0.026	0.064	0.112	0.079	0.066	0.066	0.008	0.031	0.007	0.021	0.019
**26**	0.025	0.008	0.013	0.004	0.032	0.038	0.066	0.145	0.136	0.073	0.103	0.109	0.054	0.014	0.032	0.012	0.016	0.016	0.015	0.044
**27**	0.012	0.007	0.019	0.010	0.031	0.027	0.035	0.165	0.156	0.144	0.087	0.066	0.046	0.028	0.029	0.022	0.018	0.020	0.008	0.019
**28**	0.011	0.010	0.027	0.013	0.027	0.015	0.049	0.123	0.108	0.123	0.107	0.164	0.051	0.033	0.050	0.006	0.011	0.013	0.013	0.012
**29**	0.004	0.008	0.017	0.011	0.032	0.032	0.078	0.180	0.147	0.096	0.104	0.092	0.051	0.022	0.034	0.006	0.021	0.023	0.015	0.004
**30**	0.012	0.009	0.016	0.010	0.021	0.046	0.088	0.128	0.093	0.098	0.121	0.141	0.047	0.034	0.029	0.015	0.012	0.016	0.020	0.009
**31**	0.024	0.008	0.027	0.010	0.038	0.035	0.073	0.156	0.097	0.090	0.061	0.092	0.070	0.033	0.061	0.006	0.020	0.021	0.010	0.025
**32**	0.011	0.004	0.015	0.015	0.030	0.035	0.054	0.157	0.136	0.108	0.120	0.092	0.069	0.029	0.027	0.011	0.016	0.012	0.013	0.011

**Table 2:** Multiple regression analyses based on the stepwise method in first-order Markov chain.

	Model 1					Model 2				
Variable	B	SE B	β	VIF	CI	B	SE B	β	VIF	CI
0,1	-112.98	49.85	-.38*	1.00	7.03	-137.64	47.29	-.47**	1.05	6.99
0,2						147.31	60.48	.39*	1.05	9.61
R^2^	.12					.24				
F	5.14*					5.96**				

* *p* < 0.05

** *p* < 0.01

*** *p* < 0.001

SE = standard error, VIF = variance inflation factor, CI = condition index

Thirty-seven interval patterns with three tones that appear in all 32 sonatas were detected ([0,–1,–1], [0,–1,–3], [0,–1,0], [0,–1,2], [0,–2,–2], [0,–2,–3], [0,–2,–4], [0,–2,0], [0,–2,2], [0,–2,5], [0,–3,–2], [0,–3,–5], [0,–4,–5], [0,–5,0], [0,0,–1], [0,0,–2], [0,0,0], [0,0,1], [0,0,2], [0,0,3], [0,0,5], [0,1,–1], [0,1,–2], [0,1,–4], [0,1,0], [0,1,1], [0,1,3], [0,2,0], [0,2,3], [0,2,4], [0,3,0], [0,3,1], [0,3,2], [0,3,3], [0,4,2], [0,4,7], and [0,5,9]) (Table 3). Using these interval patterns, a multiple linear regression based on the stepwise method was carried out to predict the numbers of music based on the transitional probabilities of the 37 interval patterns in the second-order Markov chain. A significant regression equation was found (F(4,27) = 10.10, *p* = 0.0001), with an adjusted R^2^ of 0.54 (Table 4). The predicted sonata opus number is equal to 32.72–71.15 (transition of [0,–2,–4])– 50.24 (transition of [0,0,–1]) + 36.37 (transition of [0,–4,–5]) + 42.27 (transition of [0,–2,0]). The transitional probabilities of [0,–2,–4] and [0,0,–1] gradually decreased and the transitional probabilities of [0,–4,–5], and [0,–2,0] gradually increased consistently with the ascending order of the sonata opus numbers ([0,–2,–4] p = 0.011, [0,0,–1] *p* = 0.002, [0,–4,–5] *p* = 0.008, [0,–2,0] *p* = 0.020). These transitional probabilities were significant predictors of the sonata opus numbers.

**Table 3:** Transitional probabilities calculated using second-order Markov chains for each of the interval patterns.

Op.	Interval pattern																																				
	0,-5,0	0,-4,-5	0,-3,-5	0,-3,-2	0,-2,-4	0,-2,-3	0,-2,-2	0,-2,0	0,-2,2	0,-2,5	0,-1,-3	0,-1,-1	0,-1,0	0,-1,2	0,0,-2	0,0,-1	0,0,0	0,0,1	0,0,2	0,0,3	0,0,5	0,1,-4	0,1,-2	0,1,-1	0,1,0	0,1,1	0,1,3	0,2,0	0,2,3	0,2,4	0,3,0	0,3,1	0,3,2	0,3,3	0,4,2	0,4,7	0,5,9
**1**	0.035	0.035	0.026	0.080	0.247	0.351	0.079	0.139	0.016	0.016	0.397	0.052	0.314	0.033	0.151	0.166	0.337	0.036	0.068	0.056	0.044	0.017	0.088	0.008	0.237	0.052	0.275	0.326	0.224	0.161	0.045	0.309	0.197	0.096	0.077	0.359	0.109
**2**	0.127	0.037	0.044	0.104	0.361	0.358	0.042	0.041	0.019	0.009	0.462	0.087	0.192	0.076	0.058	0.092	0.425	0.089	0.049	0.070	0.028	0.037	0.017	0.025	0.110	0.040	0.217	0.122	0.344	0.333	0.018	0.301	0.147	0.086	0.150	0.478	0.311
**3**	0.022	0.086	0.074	0.123	0.304	0.322	0.025	0.153	0.028	0.009	0.292	0.067	0.431	0.034	0.153	0.437	0.218	0.105	0.009	0.009	0.031	0.033	0.113	0.023	0.192	0.060	0.246	0.201	0.308	0.237	0.066	0.095	0.079	0.021	0.088	0.302	0.180
**4**	0.120	0.063	0.059	0.189	0.241	0.366	0.105	0.056	0.026	0.007	0.254	0.061	0.346	0.163	0.142	0.211	0.350	0.039	0.022	0.051	0.010	0.023	0.037	0.028	0.118	0.048	0.180	0.225	0.164	0.196	0.133	0.232	0.058	0.019	0.130	0.178	0.218
**5**	0.200	0.067	0.045	0.083	0.279	0.357	0.065	0.041	0.014	0.046	0.346	0.049	0.251	0.083	0.115	0.182	0.252	0.057	0.073	0.035	0.035	0.011	0.037	0.015	0.075	0.060	0.247	0.268	0.242	0.328	0.067	0.233	0.193	0.040	0.118	0.235	0.057
**6**	0.138	0.101	0.026	0.171	0.290	0.309	0.113	0.067	0.015	0.044	0.330	0.099	0.306	0.161	0.040	0.125	0.509	0.070	0.114	0.006	0.042	0.065	0.062	0.024	0.173	0.078	0.270	0.320	0.238	0.142	0.110	0.280	0.022	0.038	0.271	0.086	0.056
**7**	0.033	0.096	0.121	0.102	0.261	0.348	0.085	0.048	0.027	0.030	0.322	0.038	0.332	0.049	0.124	0.162	0.379	0.059	0.048	0.028	0.045	0.026	0.037	0.014	0.086	0.067	0.166	0.263	0.282	0.184	0.044	0.213	0.088	0.013	0.066	0.166	0.157
**8**	0.060	0.235	0.122	0.122	0.271	0.369	0.120	0.061	0.012	0.014	0.339	0.073	0.186	0.071	0.174	0.249	0.257	0.064	0.117	0.008	0.042	0.025	0.058	0.025	0.089	0.100	0.277	0.197	0.378	0.177	0.370	0.162	0.078	0.019	0.057	0.092	0.081
**9**	0.027	0.050	0.010	0.038	0.330	0.424	0.049	0.065	0.013	0.016	0.353	0.024	0.301	0.105	0.014	0.149	0.577	0.028	0.060	0.005	0.028	0.044	0.006	0.034	0.088	0.106	0.156	0.134	0.384	0.274	0.115	0.204	0.124	0.027	0.130	0.145	0.022
**10**	0.014	0.074	0.125	0.197	0.244	0.316	0.112	0.059	0.019	0.016	0.382	0.087	0.320	0.044	0.170	0.134	0.404	0.032	0.036	0.018	0.029	0.050	0.036	0.028	0.084	0.062	0.279	0.125	0.393	0.409	0.073	0.236	0.491	0.018	0.320	0.160	0.021
**11**	0.217	0.067	0.075	0.048	0.288	0.316	0.046	0.129	0.022	0.013	0.291	0.038	0.516	0.028	0.162	0.162	0.168	0.117	0.140	0.067	0.022	0.046	0.067	0.024	0.198	0.022	0.189	0.206	0.319	0.273	0.118	0.179	0.087	0.004	0.097	0.306	0.244
**12**	0.320	0.130	0.016	0.109	0.280	0.220	0.059	0.227	0.006	0.006	0.270	0.137	0.303	0.008	0.037	0.078	0.663	0.058	0.014	0.010	0.023	0.007	0.067	0.060	0.142	0.149	0.205	0.271	0.242	0.195	0.354	0.062	0.062	0.062	0.140	0.107	0.057
**13**	0.149	0.090	0.187	0.043	0.326	0.306	0.061	0.035	0.048	0.016	0.273	0.059	0.378	0.113	0.075	0.109	0.547	0.015	0.026	0.030	0.026	0.062	0.044	0.022	0.220	0.093	0.233	0.058	0.368	0.437	0.317	0.174	0.096	0.030	0.055	0.315	0.105
**14**	0.393	0.055	0.022	0.045	0.190	0.186	0.121	0.152	0.009	0.013	0.383	0.173	0.093	0.080	0.016	0.101	0.474	0.035	0.019	0.044	0.052	0.018	0.043	0.030	0.098	0.037	0.293	0.157	0.245	0.301	0.026	0.055	0.035	0.040	0.011	0.242	0.157
**15**	0.076	0.119	0.064	0.165	0.335	0.394	0.058	0.039	0.005	0.025	0.331	0.031	0.310	0.122	0.075	0.180	0.418	0.049	0.042	0.020	0.042	0.039	0.116	0.020	0.217	0.092	0.208	0.214	0.350	0.185	0.060	0.352	0.086	0.004	0.147	0.431	0.319
**16**	0.228	0.113	0.061	0.165	0.256	0.321	0.041	0.093	0.068	0.004	0.220	0.020	0.452	0.070	0.040	0.135	0.501	0.027	0.036	0.009	0.007	0.068	0.072	0.016	0.113	0.057	0.195	0.376	0.228	0.235	0.160	0.321	0.153	0.059	0.203	0.211	0.129
**17**	0.142	0.010	0.044	0.120	0.162	0.270	0.220	0.008	0.017	0.012	0.167	0.104	0.379	0.016	0.124	0.162	0.124	0.143	0.038	0.054	0.011	0.023	0.046	0.005	0.190	0.181	0.151	0.111	0.281	0.190	0.118	0.034	0.034	0.050	0.026	0.123	0.090
**18**	0.074	0.059	0.066	0.189	0.199	0.306	0.071	0.036	0.087	0.005	0.284	0.030	0.362	0.074	0.067	0.040	0.325	0.056	0.016	0.078	0.102	0.072	0.030	0.008	0.089	0.065	0.295	0.168	0.245	0.287	0.269	0.138	0.034	0.061	0.137	0.133	0.188
**19**	0.148	0.361	0.133	0.083	0.251	0.345	0.119	0.149	0.009	0.004	0.270	0.054	0.446	0.108	0.103	0.179	0.342	0.087	0.022	0.033	0.092	0.065	0.112	0.014	0.093	0.056	0.195	0.101	0.323	0.272	0.071	0.464	0.089	0.196	0.256	0.179	0.052
**20**	0.256	0.050	0.260	0.078	0.227	0.206	0.115	0.097	0.009	0.040	0.328	0.187	0.201	0.037	0.161	0.082	0.382	0.064	0.061	0.021	0.014	0.025	0.062	0.086	0.148	0.259	0.272	0.181	0.156	0.281	0.186	0.339	0.153	0.085	0.094	0.688	0.300
**21**	0.119	0.104	0.063	0.057	0.259	0.334	0.012	0.056	0.030	0.004	0.378	0.005	0.419	0.077	0.026	0.029	0.529	0.036	0.049	0.016	0.021	0.038	0.062	0.038	0.203	0.032	0.243	0.186	0.346	0.232	0.333	0.086	0.099	0.036	0.168	0.220	0.213
**22**	0.211	0.010	0.016	0.219	0.221	0.256	0.010	0.241	0.030	0.005	0.155	0.028	0.641	0.028	0.008	0.050	0.661	0.021	0.008	0.017	0.033	0.020	0.234	0.051	0.153	0.047	0.166	0.232	0.202	0.228	0.056	0.100	0.061	0.017	0.039	0.455	0.089
**23**	0.257	0.114	0.047	0.107	0.243	0.329	0.015	0.195	0.026	0.022	0.382	0.030	0.234	0.064	0.055	0.069	0.547	0.040	0.024	0.024	0.014	0.119	0.104	0.018	0.272	0.056	0.244	0.538	0.168	0.091	0.159	0.020	0.022	0.022	0.048	0.273	0.161
**24**	0.234	0.082	0.052	0.060	0.239	0.346	0.057	0.088	0.025	0.016	0.252	0.025	0.344	0.064	0.048	0.043	0.080	0.027	0.059	0.207	0.053	0.070	0.056	0.007	0.178	0.021	0.178	0.239	0.187	0.113	0.211	0.058	0.063	0.126	0.261	0.136	0.087
**25**	0.341	0.082	0.077	0.138	0.278	0.290	0.034	0.130	0.025	0.025	0.411	0.056	0.250	0.117	0.147	0.015	0.309	0.074	0.059	0.132	0.044	0.103	0.085	0.006	0.194	0.067	0.194	0.324	0.201	0.294	0.293	0.044	0.073	0.015	0.053	0.140	0.118
**26**	0.031	0.365	0.182	0.045	0.288	0.315	0.053	0.094	0.016	0.034	0.218	0.068	0.347	0.119	0.136	0.127	0.245	0.068	0.055	0.041	0.018	0.068	0.068	0.010	0.184	0.045	0.355	0.221	0.279	0.324	0.123	0.167	0.148	0.086	0.098	0.244	0.133
**27**	0.121	0.314	0.091	0.015	0.243	0.327	0.136	0.068	0.029	0.026	0.331	0.137	0.232	0.017	0.125	0.103	0.303	0.052	0.063	0.033	0.066	0.006	0.025	0.006	0.148	0.093	0.185	0.252	0.285	0.163	0.253	0.207	0.184	0.103	0.321	0.075	0.037
**28**	0.025	0.152	0.171	0.144	0.193	0.196	0.125	0.280	0.005	0.005	0.207	0.127	0.214	0.025	0.165	0.176	0.230	0.057	0.087	0.022	0.030	0.066	0.060	0.006	0.160	0.038	0.352	0.154	0.292	0.282	0.078	0.149	0.045	0.065	0.082	0.286	0.108
**29**	0.130	0.163	0.095	0.098	0.273	0.299	0.096	0.108	0.034	0.003	0.298	0.098	0.207	0.072	0.099	0.110	0.269	0.049	0.063	0.026	0.050	0.028	0.036	0.027	0.142	0.081	0.286	0.217	0.292	0.247	0.123	0.185	0.080	0.164	0.149	0.109	0.085
**30**	0.232	0.050	0.035	0.126	0.242	0.271	0.077	0.218	0.009	0.018	0.205	0.094	0.307	0.070	0.070	0.120	0.291	0.089	0.062	0.023	0.031	0.025	0.079	0.028	0.142	0.054	0.290	0.224	0.259	0.243	0.288	0.144	0.064	0.048	0.256	0.067	0.104
**31**	0.266	0.023	0.055	0.105	0.187	0.238	0.054	0.223	0.018	0.016	0.276	0.046	0.163	0.075	0.114	0.145	0.405	0.064	0.123	0.023	0.041	0.020	0.132	0.020	0.113	0.066	0.305	0.176	0.194	0.154	0.075	0.345	0.144	0.029	0.207	0.146	0.060
**32**	0.126	0.100	0.070	0.098	0.170	0.317	0.053	0.166	0.032	0.013	0.262	0.065	0.376	0.077	0.114	0.133	0.369	0.084	0.044	0.019	0.049	0.023	0.029	0.027	0.260	0.061	0.273	0.386	0.181	0.192	0.095	0.167	0.145	0.091	0.190	0.216	0.093

**Table 4:** Multiple regression analyses based on the stepwise method in second-order Markov chain.

	Model 1					Model 2				
Variable	B	SE B	β	VIF	CI	B	SE B	β	VIF	CI
0,-2,-4	-97.49	30.99	-.50**	1.00	10.92	-85.50	28.98	-.44**	1.03	4.06
0,0,-1						-44.71	17.85	-.37*	1.03	13.01
0,-4,-5										
0,-2,0										
R^2^	.22					.34				
F	9.90**					8.96**				
	Model 3					Model 4				
Variable	B	SE B	β	VIF	CI	B	SE B	β	VIF	CI
0,-2,-4	-94.13	26.49	-.48**	1.04	3.27	-71.15	26.08	-.36*	1.20	3.40
0,0,-1	-48.76	16.26	-.40**	1.04	4.58	-50.24	14.97	-.42**	1.04	3.95
0,-4,-5	37.33	13.89	.36*	1.03	14.50	36.37	12.78	.35**	1.03	5.29
0,-2,0						42.27	17.10	.32*	1.15	18.17
R^2^	.46					.54				
F	9.66***					10.10***				

* *p* < 0.05

** *p* < 0.01

*** *p* < 0.001

SE = standard error, VIF = variance inflation factor, CI **=** condition index

Twelve interval patterns with four tones that appear in all 32 sonatas were detected ([0,–1,–3,–5], [0,–1,0,–1], [0,–1,0,2], [0,–2,–3,–2], [0,–2,–3,–5], [0,–2,–4,–5], [0,0,0,0 ], [0,1,3,1 ], [0,1,3,5], [0,2,0,–1], [0,2,3,5], and [0,2,4,5]) (Table 5). Using these interval patterns, a multiple linear regression based on the stepwise method was carried out to predict the sonata opus numbers based on the transitional probabilities of the 12 interval patterns in the third-order Markov chain. A significant regression equation was found (F(2,29) = 9.25, *p* = 0.001), with an adjusted R^2^ of 0.35 (Table 6). The predicted sonata opus number is equal to 39.12–28.81 (transition of [0,2,0,–1])– 24.82 (transition of [0,2,4,5]). The transitional probabilities of [0,2,0,–1] and [0,2,4,5] gradually decreased consistently with the ascending order of the sonata opus numbers ([0,2,0,–1] *p* = 0.003, [0,2,4,5] *p* = 0.011). These transitional probabilities were significant predictors of the sonata opus numbers.

**Table 5:** Transitional probabilities calculated using third-order Markov chains for each of the interval patterns.

Op.	Interval pattern											
	0,-1,-3,-5	0,-1,0,-1	0,-1,0,2	0,-2,-3,-2	0,-2,-3,-5	0,-2,-4,-5	0,0,0,0	0,1,3,1	0,1,3,5	0,2,0,-1	0,2,3,5	0,2,4,5
**1**	0.385	0.186	0.342	0.311	0.437	0.609	0.421	0.360	0.220	0.346	0.347	0.692
**2**	0.723	0.179	0.250	0.140	0.588	0.609	0.435	0.177	0.554	0.689	0.484	0.650
**3**	0.631	0.138	0.351	0.376	0.471	0.574	0.500	0.220	0.372	0.407	0.348	0.607
**4**	0.592	0.220	0.190	0.348	0.312	0.665	0.387	0.236	0.439	0.484	0.353	0.407
**5**	0.355	0.080	0.193	0.213	0.471	0.579	0.308	0.288	0.500	0.453	0.625	0.523
**6**	0.553	0.263	0.298	0.236	0.338	0.532	0.582	0.470	0.120	0.474	0.319	0.535
**7**	0.403	0.099	0.249	0.245	0.351	0.429	0.679	0.298	0.351	0.600	0.280	0.449
**8**	0.439	0.150	0.475	0.199	0.414	0.647	0.507	0.340	0.180	0.345	0.117	0.250
**9**	0.644	0.163	0.244	0.191	0.412	0.667	0.780	0.200	0.520	0.500	0.333	0.556
**10**	0.514	0.250	0.398	0.313	0.343	0.471	0.563	0.086	0.633	0.622	0.461	0.762
**11**	0.594	0.318	0.259	0.288	0.479	0.663	0.400	0.161	0.427	0.270	0.297	0.568
**12**	0.785	0.356	0.178	0.183	0.606	0.611	0.788	0.036	0.309	0.047	0.193	0.674
**13**	0.677	0.456	0.222	0.221	0.295	0.564	0.710	0.132	0.679	0.455	0.429	0.639
**14**	0.242	0.200	0.333	0.070	0.349	0.136	0.644	0.063	0.708	0.206	0.755	0.615
**15**	0.673	0.253	0.222	0.201	0.582	0.624	0.586	0.379	0.221	0.346	0.353	0.644
**16**	0.732	0.107	0.348	0.554	0.338	0.390	0.758	0.427	0.266	0.415	0.225	0.467
**17**	0.394	0.429	0.112	0.277	0.323	0.615	0.261	0.197	0.242	0.176	0.349	0.655
**18**	0.506	0.227	0.464	0.342	0.375	0.731	0.782	0.129	0.548	0.393	0.483	0.567
**19**	0.509	0.033	0.154	0.247	0.444	0.508	0.460	0.214	0.333	0.313	0.275	0.419
**20**	0.727	0.333	0.148	0.136	0.500	0.425	0.206	0.091	0.773	0.207	0.640	0.467
**21**	0.424	0.215	0.301	0.395	0.469	0.595	0.759	0.177	0.310	0.294	0.316	0.575
**22**	0.571	0.147	0.147	0.549	0.412	0.545	0.788	0.490	0.429	0.302	0.413	0.462
**23**	0.435	0.318	0.288	0.274	0.523	0.438	0.592	0.417	0.135	0.505	0.303	0.333
**24**	0.563	0.113	0.227	0.364	0.318	0.566	0.571	0.078	0.216	0.382	0.209	0.154
**25**	0.595	0.044	0.156	0.191	0.500	0.656	0.048	0.188	0.313	0.074	0.271	0.407
**26**	0.700	0.301	0.252	0.159	0.536	0.635	0.611	0.155	0.700	0.192	0.598	0.579
**27**	0.406	0.162	0.132	0.149	0.634	0.533	0.561	0.367	0.233	0.355	0.171	0.100
**28**	0.552	0.101	0.275	0.375	0.278	0.296	0.365	0.188	0.446	0.303	0.486	0.532
**29**	0.533	0.157	0.220	0.281	0.343	0.504	0.531	0.284	0.279	0.245	0.404	0.515
**30**	0.600	0.149	0.338	0.185	0.196	0.500	0.560	0.130	0.435	0.169	0.427	0.456
**31**	0.364	0.256	0.487	0.272	0.272	0.611	0.557	0.239	0.304	0.175	0.318	0.429
**32**	0.324	0.319	0.353	0.444	0.222	0.509	0.471	0.462	0.146	0.482	0.273	0.443

**Table 6:** Multiple regression analyses based on the stepwise method in third-order Markov chain.

	Model 1					Model 2				
Variable	B	SE B	β	VIF	CI	B	SE B	β	VIF	CI
0,2,0,-1	-29.53	9.73	-.49**	1.00	4.83	-28.81	8.84	-.47**	1.00	4.82
0,2,4,5						-24.82	9.16	-.39*	1.00	9.00
R^2^	.21					.35				
F	9.22**					9.25**				

* *p* < 0.05

** *p* < 0.01

*** *p* < 0.001

SE = standard error, VIF = variance inflation factor, CI **=** condition index

Three interval patterns with five tones that appear in all 32 sonatas were detected ([0,–1,–3,–5,–6], [0,–2,–3,–5,–7], and [0,–2,–4,–5,–7]) (Table 7). Using these interval patterns, a multiple linear regression based on the stepwise method was carried out to predict the sonata opus numbers based on the transitional probabilities of the three interval patterns in the fourth-order Markov chain. A significant regression equation was found (F(1,30) = 6.65, *p* = 0.015), with an adjusted R^2^ of 0.15 (Table 8). The predicted sonata opus number is equal to 27.13–19.27 (transition of [0,–2,–3,–5,–7]). The transitional probabilities gradually decreased consistently with the ascending order of the sonata opus numbers (*p* = 0.015). The transitional probabilities were significant predictors of the sonata opus numbers. To understand how the transitional probabilities of [0,–2,–3,–5,–7] were shifted in more detail, the transition matrices based on the fourth-order Markov chain of P(X|0, -2, -3, -5) in the first (No.1) and last (No.32) sonatas are shown in Table 9. Furthermore, the representative phrases that transitioned with the highest probability in the first and last sonata were decoded as music scores (Fig 1).

**Table 7:** Transitional probabilities calculated using fourth-order Markov chains for each of the interval patterns.

Op.	Interval pattern		
	0,-1,-3,-5,-6	0,-2,-3,-5,-7	0,-2,-4,-5,-7
**1**	0.380	0.567	0.695
**2**	0.500	0.739	0.650
**3**	0.475	0.712	0.675
**4**	0.609	0.696	0.360
**5**	0.535	0.356	0.729
**6**	0.353	0.740	0.473
**7**	0.296	0.493	0.448
**8**	0.754	0.507	0.593
**9**	0.585	0.648	0.485
**10**	0.370	0.717	0.531
**11**	0.380	0.567	0.695
**12**	0.431	0.907	0.727
**13**	0.455	0.607	0.281
**14**	0.133	0.667	0.167
**15**	0.458	0.702	0.603
**16**	0.322	0.813	0.522
**17**	0.536	0.048	0.792
**18**	0.487	0.556	0.491
**19**	0.357	0.417	0.533
**20**	0.281	0.788	0.710
**21**	0.571	0.537	0.580
**22**	0.438	0.571	0.500
**23**	0.404	0.333	0.603
**24**	0.600	0.571	0.419
**25**	0.682	0.489	0.559
**26**	0.508	0.743	0.700
**27**	0.410	0.476	0.575
**28**	0.243	0.200	0.238
**29**	0.369	0.654	0.504
**30**	0.467	0.556	0.268
**31**	0.167	0.160	0.227
**32**	0.565	0.114	0.130

**Table 8:** Multiple regression analyses based on the stepwise method in fourth-order Markov chain.

	Model 1				
Variable	B	SE B	β	VIF	CI
0,-2,-3,-5,-7	-19.27	7.47	-.43*	1.00	5.58
R^2^	.15				
F	6.65*				

* *p* < 0.05

** *p* < 0.01

*** *p* < 0.001

SE = standard error, VIF = variance inflation factor, CI **=** condition index

**Table 9:** Transition matrices based on the fourth-order Markov chain (P(X|0, -2, -3, -5)).

Piano Sonata No.1 in F minor, Op.2-1												
	-10	-9	-7	-6	-5	-3	-2	2	3	7	10	15
0,-2,-3,-5	0.072	0	**0.567**	0.155	0.041	0.113	0.01	0	0.01	0.021	0.01	0
Piano Sonata No.32 in C minor, Op.111												
	-10	-9	-7	-6	-5	-3	-2	2	3	7	10	15
0,-2,-3,-5	0.023	0.023	0.114	0.136	0.068	0.136	**0.227**	0.045	0.159	0	0	0.068

One interval pattern with six tones that appeared in all 32 sonatas was detected (0,–2,–4,–5,–7,–9) (Table 10). Using these interval patterns, a multiple linear regression based on the stepwise method was carried out to predict the sonata opus numbers based on the transitional probabilities of the interval patterns in the fifth-order Markov chain. No significant regression equation was detected, however. No sixth- or higher-order interval patterns appeared in all 32 sonatas.

**Table 10:** Transitional probabilities calculated using first-order Markov chains for each of the interval patterns.

Op.	Transition pattern Transition pattern
	0,-2,-4,-5,-7,-9
**1**	0.591
**2**	0.692
**3**	0.753
**4**	0.725
**5**	0.373
**6**	0.771
**7**	0.633
**8**	0.529
**9**	0.848
**10**	0.769
**11**	0.627
**12**	0.925
**13**	0.875
**14**	1.000
**15**	0.660
**16**	0.639
**17**	0.053
**18**	0.464
**19**	0.625
**20**	0.682
**21**	0.667
**22**	0.833
**23**	0.468
**24**	0.778
**25**	0.424
**26**	0.839
**27**	0.739
**28**	0.200
**29**	0.671
**30**	0.636
**31**	0.200
**32**	0.429

## 4. Discussion

### 4.1. Psychological relationships between variation of statistical structure in music and implicit knowledge

A tone with a higher transitional probability of sequences in a musical score may be one that a composer is more likely to choose compared to other tones with lower transitional probability [20–23,58–60]. Thus, the transitional probability matrix calculated from music may represent the characteristics of a composer’s statistical knowledge by which a forthcoming tone is implicitly defined. The present study aimed to investigate how the statistical structures in music vary over a composer’s lifetime. I hypothesized that there might be two types of time-course variations: transitional probabilities that gradually decrease and those that gradually increase, consistent with the order of the sonata opus number. If so, these findings suggest that statistical knowledge of music, which are generally considered implicit phenomenon, gradually shifts over a composer’s lifetime.

Consistent with the order of the sonata opus numbers, the transitional probabilities of [0,1], [0,–2,–4], [0,0,–1], [0,2,0,–1], [0,2,4,5], and [0,–2,–3,–5,–7] gradually decreased, and those of [0,2], [0,–4,–5], and [0,–2,0] gradually increased. All of these transitions may be musical universal. The examples of each transition were shown in Table 11. The transition matrices of P(X|0, -2, -3, -5) in the first (No.1) and last sonatas (No.32) are shown in Table 10. The representative transition of [0,–2,–3,–5,–7] in No.1 was decoded as musical scores in Fig 1A. In these familiar sequences, decreasing transitional probabilities were detected more frequently than increasing transitional probabilities. This finding suggests that, in the later period of his life, Beethoven composed statistically novel music in which predictable and familiar transitions did not appear compared to the earlier periods. The sequences with 5 tones based on fourth-order transitional probabilities were the longest in the transitions that showed significance, suggesting that the time-course variation of statistical structure that could implicitly reflect strategy of music composition may depend on until fourth-order transitional probabilities. According to the previous neurophysiological study on statistical learning, fourth-order transitional probabilities in auditory sequences modulate prediction in human’s brain [61]. Furthermore, they suggested that, higher-order transitional probabilities could modulate learner’s prediction when they learn more difficult statistical structure. These findings may suggest that the findings of the present study are in part related to prediction and expectation in the composer, although this study could not detect difference among the orders of transitional probability. On the other hand, the only interval patterns that appear in all 32 sonatas were used to verify the variation of transitional probabilities of the familiar sequences in the 32 sonatas. In total, there are more than several billion kinds of interval patterns in music. Thus, it is difficult to define the statistical significance when we target all of the interval patterns. Future study will need to investigate interval patterns that do not appear in some sonatas.

**Table 11:** The examples of transitions in which transitional probabilities were gradually increased and decreased.

Variation	Transition	Number of notes	Examples in C major
Decrease	0,1	III,IV	E,F
		VII,I	B,C
	0,-2,-4	I,II,III	C,D,E
		IV,V,VI	F,G,A
		V,VI,VII	G,A,B
	0,0,-1	I,I,VII	C,C,B
		IV,IV,III	F,F,E
	0,2,0,-1	I,II,I,VII	C,D,C,B
		IV,V,IV,III	F,G,F,E
	0,2,4,5	I,II,III,IV	C,D,E,F
		V,VI,VII,I	G,A,B,C
	0,-2,-3,-5,-7	V,IV,III,II,I	G,F,E,D,C
		II,I,VII,VI,V	D,C,B,A,G
Increase	0,2	I,II	C,D
		II,III	D,E
		IV,V	F,G
		V,VI	G,A
		VI,VII	A,B
	0,-4,-5	III,I,VII	E,C,B
		VI,IV,III	A,F,E
	0,-2,0	II,I,II	D,C,D
		III,II,III	E,D,E
		V,IV,V	G,F,G
		VI,V,VI	A,G,A
		VII,VI,VII	B,A,B

To understand how the highest-order (i.e., fourth-order) transitional probabilities of [0,–2,–3,–5,–7] shifted in more detail, the transition matrices based on the fourth-order Markov chain of P(X|0, -2, -3, -5) in the first (No.1) and last (No.32) sonatas were investigated (Table 10). In the transition matrices of Sonata No.1 (Table 10), the distribution of transitional probabilities was biased: the transitional probability of [0,–2,–3,–5,–7] was markedly higher than that of any other transitional probabilities. In contrast, in the transition matrices of Sonata No.32 (Table 10), the transitional probabilities were equally distributed compared to the transition matrices of Sonata No.1: there was no obvious highest transitional probability. The representative phrases with the highest transitional probability in Sonatas No.1 and No.32 were decoded as musical scores in Fig 1A and 1B. These findings are consistent with the hypothesis that, in the last sonata, Beethoven tried several composition methods in which he avoided familiar interval patterns. The previous studies indicated that statistical learning contributes to many types of mental representation in music: the comprehension and production of music [62], intuitive decision-making [63,64] and creativity involved in musical composition [15]. The several studies also suggest that, compared to language [65], musical representation including tonality is mainly formed by a tacit knowledge [66–72]. Furthermore, the neurophysiological studies indicates that the distribution of pitch frequency that mainly forms musical melody, rather than other acoustic features such as formant frequencies that play an important role in forming speech sounds instead of melody, facilitate auditory statistical learning in human’s brain [22]. Thus, it is widely accepted that statistical knowledge is tied to musical expression such as composition, playing, and creativity [12–17], even if learners cannot realize the acquired knowledge due to implicitness of statistical learning [21]. Given these previous findings, the results of the present study may suggest that the time-course variations of statistical structures in music represent the time-course variations of a composer’s statistical knowledge.

The previous studies demonstrated that the transitional probabilities of relative-pitch patterns could be learned [21,22,73]. Based on these findings, this study analyzed pitch-interval patterns but not absolute-pitch pattern like the methodologies of the previous studies [43,56] in order to eliminate the effects of the change of key on interval patterns. In other words, the present study verifies statistical distribution of relative- but not absolute-pitch structures.

### 4.2. Musicological relationships between Beethoven’s lifetime and the statistical variations of music

The numbers of Beethoven’s Piano Sonata indicate the order in which the music was published, not when it was composed. Although most of the numbers are consistent with the order in which the music was composed, Piano Sonata No. 19 in G minor and Piano Sonata No. 20 in G major, which were published around 1805, are considered to have been composed a decade earlier, between 1795 and 1798 [74,75]. This means that Piano Sonata No.19 and No.20 may have been written around the time that Beethoven composed Piano Sonata No.3 and No.4. The specific time of composition remains controversial. Therefore, in the present study, the time courses in which the music was published are used, as they are clearer than the time courses in which the music was composed. The present study revealed the time-course variations of statistical structures in Beethoven’s music over his lifetime. In other words, the statistically novel music was composed during Beethoven’s significant life events in late periods when he was already troubled by deafness and irritability brought on by chronic abdominal pain and were considered to show his personal expression and intellectual depth [51,52]. According to the previous studies, statistical learning could modulate strategies of musical composition, and the musical training and experience is associated with cognitive model of probabilistic structure in music involved in statistical learning [43,12–18]. The neurophysiological studies also demonstrated that musical training modulates the abilities of statistical learning [76,77,78]. These previous findings may suggest that musical experience allows a composer to change the strategies of musical composition based on the acquired statistical knowledge over his/her lifetime. The results of time-course variation of statistical structure in the present study may imply that, in later compositional periods, because Beethoven experienced a lot of composition strategies and explored new directions, he might compose statistically novel music in which predictable transitions did not appear compared to that of earlier periods. According to the previous studies, corpus analyses can detect the historical characteristics of music and in part distinguish them based on the era (e.g., [37,40,79]), suggesting that variation of statistical structure can be detected over long periods of time. The present corpus study also detected the variation of characteristics within a composer in his/her lifetime. It is of note that the present study did not directly demonstrate that the implicit statistical knowledge of music varied, as only the statistics of musical scores were analyzed. This suggests that there are other possible explanations for the results in the present study. For instance, it might be part of his plan to compose the sonatas from familiar to increasingly unfamiliar based on the statistical structure of music. It cannot be excluded the possibility that the findings in the present study do not necessarily reflect that Beethoven’s statistical knowledge changed. The human’s ability to generate random sequences of numbers [80] has been associated with creativity in human [81]. In addition, to understand variation of transitional probability of more general phrase that is consistently used by Beethoven, the present study only analyzed the sequences that appeared in all pieces of music. One of the reasons is that the phrases that do not appear in all sonatas and showed transitional probability of 0% in some pieces of music is difficult to define composer’s knowledge. Compared with these phrases, the consistently appeared phrases may depend on his knowledge. However, there may be another specific variation in phrases that showed transitional probability of 0% in some pieces of music. Future study should investigate the effects of statistical learning and knowledge of all types of phrase on music compositions. The present study shed new light on novel methodologies that may be able to evaluate the time-course variation of composer’s implicit knowledge using musical scores.

## Supporting information

S1 Table The coded sequence in each piece of music. (XLSX)
